# Appointment and Promotion of Faculty in Medical and Dental Institutions: Understanding the Criterion for Assessment of Research Articles

**DOI:** 10.12669/pjms.36.4.2344

**Published:** 2020

**Authors:** Fazal Ghani

**Affiliations:** Prof. Dr. Fazal Ghani, PhD, FDSRCPSGlasg, MSc, BDS, BSc. Professor & Dean Postgraduate Dental Studies, Peshawar Dental College, Warsak Road, Peshawar 25160, Pakistan. Email: fazalg55@hotmail.com

An important aspect ensuring quality education and training in a medical and dental institution is the credential of its faculty. So far when it comes to the faculty appointment or promotion, it is quite clear that neither the proposed criteria have been followed nor it has received the attention it deserves. Three criteria are currently used for the appointment and promotion of the faculty in medical including dental institutions. These include; relevant postgraduate qualification, length of relevant teaching experience and the minimum required number of relevant original research publications. All these criteria are very important for ensuring the culture and environment for quality education, training and research. However, when it comes to the evaluation / assessment of the research publications, the relevant aspects are seldom or inappropriately followed. Important aspects to consider when determining the relevance and quality of research articles include;

1. Relevance to the specialty.
2. Contribution and authorship and sequence of a faculty among the listed authors.
3. Quality of research presented in the article.

Looking at and assessing all these aspects of research articles is not easy and hence require some deliberations.

## Relevance of Research Article

There are simple cases in which it is not too difficult to assess the relevance of a research article to fit in a particular and a distinct specialty or discipline. However, in many cases there is an indication of dishonesty and lack of integrity on the part of authors listed in a research article and hence making evaluation becomes difficult. Many of us find it difficult regarding how and where to draw lines between two or more specialties and to determine who should do what and what not. Furthermore, the increasing popularity and emphasis of the need for collaboration between researchers from multiple specialties further complicates the knowing of relevancy of a research title or article to fit into the specialties of some listed as authors. Hence to determine where a researcher could qualify as relevant author or in other words for an article to fit in the specialty or discipline of the author (s) and of the justification for involvement of an author in the conduct and publication of that research, requires special expertise.

So far, this has been done by the so called experts of the local promotion / selection committees with guidance from the Pakistan Medical and Dental Council (PMDC) but unfortunately in the most inappropriate manner. If a scrutiny of the considered articles for the promotion of medical / dental faculty in any medical institution is performed, most of us will be extremely surprised to know about the obvious malpractice of considering irrelevant articles. One will surely see abundant examples of mixed and different specialty / subject authors in one article. Those to whom this task is delegated, need to be more vigilant and expert so as to properly determine this aspect of a research article.

## Contribution, Authorship and Sequence of the Listed Authors in a Research Article

At present, the best-known guide for deciding authorial credit is the revised Vancouver Convention of the International Council of Medical Journal Editors (ICMJE 2018).^1^ The rules given are however, not helpful in resolving cases of authors’ disputes.^2^ According to Materials Research Society (MRS)^3^ and American Physical Society (APS),^4^ authorship in a research publication is limited to those who have made a significant scientific contribution to the concept, design, execution, or interpretation of the research study. Some scientific journals have adopted the “Contributor Roles Taxonomy” (CRediT) system,^5^ which requires the lead authors to provide an accurate summary of each author’s contribution to the 14 distinct areas deemed relevant to authorship; Conceptualization, Data curation, Formal analysis, Funding acquisition, Investigation, Devising methodology, Project administration, Contribution of resources, Development of software, Supervision, Validation, Visualization, Manuscript writing and Review draft and Editing.

Traditionally, it has been the journals asking the corresponding author to provide for the sequence and contribution of each of the listed authors including their signatures and consent. No doubt, the ’Author contributions’ statement is a valuable tool for appreciating the efforts of individual scientists towards the publication of a paper. However, it is not appropriate for the editor to establish author list and sequence. In fact, this is the responsibility of the concerned scientists and their institutions. Furthermore, even with the best intention, it has never been easy to compare the relative values of intellectual and practical contributions to research and of the article arising from it. For instance, it seems very difficult for anyone to consider one author more worthy or as principal author who had come up with the idea for the key experiment than the one who carried out the bulk of the experiment. Therefore, it is important to evaluate individual contributions carefully when compiling the author list of a scientific paper. It is even more important that it should be the institution to provide for the proof of involvement of the authors in the conducted research. Such a document to prove this could be the letters indicating the study approval by the Ethics Committees/Institutional Review Board and the research trial registration office in which the names of all listed authors of the article must be present. With these documents available as part of the appointment / promotion working paper, it will be fairly easy to see who is and who is not author and also who did more and who did less or none. Hence, it must be made mandatory that these documents be available for the submitted article(s) in which the applicant claimed authorship.

The research department of the institution or the laboratory of the Research and Development (R&D) organization, where the research had been carried can also help in providing the information regarding the sequence of authorship. Considering the obvious need for a new model of credit allocation, one solution that is proving popular is to give joint first authorship to numerous collaborators.^6^ This phenomenon was seen in just 1 % of publications in the year 2000, but raised to 8.6 % in 2009 and by 2019, the majority of papers published in some journals used joint first authorship – with 11 joint first authors listed in two papers. Of the 28 papers published in the first three issues of the Journal of Clinical Investigation in 2019, for instance, 12 listed three or more authors as co-first authors, while one paper listed nine. This further highlights the importance of providing authorship information from the institution (s) where the research had been conducted.

## Quality of Research Presented in the Article

According to McMaster University, “there are only a handful of ways to do a research study properly, but one thousand ways to do it wrong”. When it comes to assessment of the quality of a published research article, there are several aspects the appointment / promotion committee may look at. The first one is the journal itself in which the article has been published. In this regard, the impact factor of the journal and the number of times the article has been cited give a clue to the quality of the research carried out by the authors. A Publishing Analytics Company^7^ has shown that some 60% of research articles published in predatory journals didn’t attract any citations at all, and that 38% were cited just up to 10 times with less than 3% of the papers attracting more than 10 citations, and none got more than 32 citations indicating the limited interest and readership for these journals or the poor quality research published in these journals. In contrast, an analyses of a random sample of 1,000 articles published in 2014 in reputable journals indexed in the Scopus database, each of those articles had an average of around 18 citations with only 9% of the papers not cited.

An alternative to impact Factor (IF) score, the Journal Transparency Index (JTI)^8^ can also be considered. As per JTI, journals are given a one-out-of-three score if they published data availability statement, two for those requiring authors to share data (subject to exceptions) and a full three out of three if they provided enough data to enable full replication of the study. Journals also receive credit if they offer the option of peer review before any research is undertaken – a published format known as “registered reports”, used by more than 200 journals, in which assessors focus on research design, rather than end results.

While most commonly, a research article is evaluated / reviewed starting the review from its beginning till its end. However, by following this linear approach to evaluating an article, it could appear more important and impressive than the study actually could be.^9^ Therefore, it is advised to first assess the “Methods” section. This lets the assessor know about the adequacy of the scientific rigor, appropriateness of sample size, experimental design and protocols and statistical analyses before reading the story of how the authors interpreted data and what they think means in the big picture. Once the methods are found robust and solid, then the assessors continue for the results and the interpretation of the data. Finally, the Introduction and Discussion Sections are read and assessed. The Abstract is obviously read first to make sure that the manuscript falls within their area of expertise. Information related to the Ethical approval and pre-registration of the research study when mentioned in the article with proof of it given can also help ensure that methodological approaches would have been likely to be robust.

A quality research article provides an account of how its authors addressed a research question (s), the means they used to do so, what they found and how the work confirmed or contradicted existing hypotheses or generated new ones. The assessors must do their best to see that the authors have presented their hypotheses and predictions as originally intended. Preregistration of research study is being increasingly adopted across different fields as a means of preventing questionable research practices and increasing transparency.^10^ Good journals strongly support the preregistration of confirmatory research (and mandates registration for clinical trials).

However, preregistration has little value if authors fail to abide by it or do not transparently report whether their project differs from what they preregistered and why. In such cases, the authors must be asked to present their researches in person to the committee and provide links to their preregistrations, specify the date of preregistration and transparently report any deviations from the original protocol given in the article. For all deviations from the preregistered protocol, the authors need to have indicated in their article(s) how they deviated from their original plan and to explain their reason for doing so (e.g., flaw, sub-optimality, etc.). To ensure transparency, unless a preregistered analysis plan is unquestionably flawed, the authors are to be asked to also report the results of their preregistered analyses alongside the new analyses.

No research project is perfect; there are always limitations that also need to be transparently reported. It is now a requirement that all research papers include a limitations section, in which authors explain methodological and other shortcomings and explicitly acknowledge alternative interpretations of their findings. Good authors^11^ are always transparent about what they did and what they found, and the journal also must commit to publishing work that is robust, transparent and appropriately presented, even if it does not yield ‘clean’ narratives.

Lastly, it needs to be made a practice to apply and use the “Reappraised” Checklist^12^ when evaluating the integrity and quality of published research article. “Reappraised” is an acronym for 11 words including; Research governance, Ethics, Authorship, Productivity (Plausibility), Plagiarism, Research conduct, Analyses and methods, Image manipulation, Statistics and data, Errors, and Data duplication and reporting. A good journal will require that the submitted manuscript pass this checklist to be accepted for publishing. The appointment / promotion committee should also re-check to ensure that the published article is up to mark as per this checklist.
